# Blood Cd levels and carotid intima-media thickness in young adults living in Padang, Indonesia

**DOI:** 10.1186/s13104-020-05042-0

**Published:** 2020-04-06

**Authors:** Cimi Ilmiawati, Mohamad Reza, Mefri Yanni, Dina Arfiani Rusjdi

**Affiliations:** 1 Department of Pharmacology, Faculty of Medicine, Andalas University, Padang, West Sumatra Indonesia; 2 Department of Biology, Faculty of Medicine, Andalas University, Padang, West Sumatra Indonesia; 3 Department of Cardiology, Faculty of Medicine, Andalas University, Padang, West Sumatra Indonesia; 4 Department of Radiology, Faculty of Medicine, Andalas University, Padang, West Sumatra Indonesia; 5 Division of Environmental Toxicology, Department of Pharmacology, Faculty of Medicine, Andalas University, Main Campus at Limau Manis, Gedung A Lantai 1, Pauh, Padang, West Sumatra 25166 Indonesia

**Keywords:** Atherosclerosis, Cadmium, Carotid, Indonesia, Intima-medial thickness, Young adults

## Abstract

**Objective:**

Cd exposure is a non-traditional risk factor of cardiovascular disease and mortality by promoting the development of atherosclerosis. The development of atherosclerosis can be monitored non-invasively by measuring carotid intima-media thickness (CIMT). This study aimed to measure the level of blood Cd and other factors known to be associated with CIMT, measured at the segment of common carotid artery (CCA) and of internal carotid artery (ICA), in young adults from Padang, West Sumatera, Indonesia, and we analyzed whether blood Cd is a predictor of CIMT.

**Results:**

We recruited 156 subjects. Median blood Cd level was 0.61 μg/L (range 0.01–5.96 μg/L), with no difference in male compared to female subjects (Mann–Whitney U test, p = 0.60). Multiple regression analysis showed that sex is the predictor of CCA IMT (adjusted R^2^ = 0.219; β = −0.438 [95% CI − 0.662, − 0.214]; p < 0.001) and ICA IMT (adjusted R^2^ = 0.165; β = − 0.529 [95% CI − 0.761, − 0.297]; p < 0.001). Blood Cd was not a predictor of CCA IMT (adjusted R^2^ = 0.219; β = − 0.101 [95% CI − 0.257, 0.055]; p = 0.203) and ICA IMT (adjusted R^2^ = 0.165; β = − 0.055 [95% CI − 0.217, 0.107]; p = 0.503) in young adults from Padang, Indonesia.

## Introduction

Extensive use of cadmium (Cd) in multitude industries and consumer products are the source of environmental exposure to Cd [1]. Cd as a toxic metal is known to have promoting effects on murine and human atherosclerosis [2, 3] and epidemiological studies show that blood Cd is associated with the risk and incident of cardiovascular disease (CVD) and mortality [4–6]. Most of the risk factor for CVD are modifiable, therefore preventable. Environmental risk factors work through initiation or promotion of pathophysiological process associated with CVD. Despite having smaller contribution as risk factors compare to traditional risk factors, environmental risk factors become substantial when the number of populations exposed is large [7]. Research within the last decade have shown that exposure to environmental pollutant, like air pollution and toxic metals (cadmium, lead, arsenic) contributes to the development and severity of CVD [8].

Epidemiological studies have shown that blood Cd level is associated with the incident and mortality of CVD in Swedish population [4]. Blood Cd is associated with CVD risk in Korean men [5] and with carotid plaque prevalence in Swedish population [9]. Cd content of carotid plaque was found to be 50 times of that in blood and Cd exposure was correlated with subclinical atherosclerosis in middle age women from Swedia [10].

Atherosclerosis is a slow but progressive vascular change beginning at young age and plays an important role in the pathogenesis of CVD, including hypertension, ischemic heart disease, peripheral arterial disease and stroke [11], the number one killer in developing country [12]. The development of atherosclerosis can be monitored by non-invasive method by measuring the intima-media thickness (IMT) of major superficial arteries, like carotid and femoral arteries.

There are many studies showings that environmental Cd exposure is associated with atherosclerosis in developed countries, however there is still limited studies in developing countries. Considering high CVD prevalence and different pattern of environmental pollutant exposure in Indonesia as a developing country compared to those of developed country, it is essential to conduct a Cd biomonitoring study in young adults from Indonesia. In the current study, we measured the level of blood Cd and other factors known to be associated with carotid intima-media thickness (CIMT) in young adults from Padang, West Sumatera, Indonesia, and we analyzed whether blood Cd is a predictor of CIMT.

## Main text

### Subjects and methods

#### Study subjects

The participants were 156 young adults, enrolled as university students, aged 18–24 living in Padang, West Sumatera, Indonesia. Subjects were invited through the student’s body organization to participate in the group health examinations for the investigation of cadmium exposure as a vascular toxicant. Subjects with history of cardiovascular disease were excluded. All subjects signed the appropriate informed consent. The study was approved by the Ethics Committee of Faculty of Medicine Andalas University (No. 350/KEP/FK/2018) and was conducted in line with the Declaration of Helsinki.

#### Cd determination in blood samples

Blood Cd was analyzed by Prodia Industrial Toxicology Laboratory (Jakarta, Indonesia) using Agilent 7700 inductively coupled plasma-mass spectrometry (ICP-MS). Blood Cd was analyzed from whole blood sample according to the method by CDC (CDC, 2011) with some modifications. The limit of detection (LOD) for Cd in blood was 0.004 μg/L and the limit of quantification (LOQ) was 0.015 μg/L. No sample was below LOQ. The level of analytical accuracy was 84.2–89.8% and the level of analytical precision was 7.8–8.7%.

#### Measurement of blood pressure

Systolic and diastolic blood pressures were measured with a digital monitor (Omron HEM-7120, Japan). Subjects were seated for five minutes before the measurement began. Blood pressure were taken three times, the average value of the last two measurements were used for analysis [13]. No regular use of antihypertensive drugs was reported. Hypertension was defined as systolic blood pressure ≥ 130 mmHg and diastolic blood pressure ≥ 80 mmHg [14].

#### Measurement of CIMT

CIMT of common carotid artery (CCA) and internal carotid artery (ICA) was measured by ultrasonography of the left and right carotid artery according to the published method [15]. CIMT measurement was available for all subjects.

#### Laboratory methods

Blood samples were drawn in non-fasting subjects and glucose, hemoglobin (Hb), triglycerides, total cholesterol, low-density lipoprotein cholesterol (LDL-cholesterol), and high-density lipoprotein cholesterol (HDL-cholesterol) were determined by standard methods. Cd bioavailability is affected by low iron status [16], therefore we determined Hb level in our subjects. Non-fasting blood glucose was measured in sera using enzymatic photometric test (DiaSys, Germany). All analyses were carried out at the Biochemistry Laboratory of Faculty of Medicine Andalas University.

#### Covariates

Factors potentially affecting CIMT included in statistical analyses were: (1) Age; (2) Sex; (3) Body mass index (BMI); (4) Parental education; (5) Exposure to cigarette smoke; (6) Systolic blood pressure; (7) Serum total cholesterol; (8) Serum HDL-cholesterol; (9) Haemoglobin; and (10) Blood glucose level. Body weight and height were measured to the nearest 0.1 kg and 0.1 cm, respectively, with subject wearing light clothing and no shoes. BMI was calculated as body weight (kg) divided by square of body height (m^2^).

Age (in year), smoking habit (never, former, current), secondhand smoking exposure (never, sometimes, frequently, always), alcohol intake (yes or no), parental education (without college education, college education, postgraduate education), parental monthly income (< 3, 310, > 10 million Indonesian rupiah), residence close to a metal workshop or industry (yes or no) were obtained from an 2-page questionnaire.

#### Statistical analysis

The distribution of blood Cd is not normal and therefore expressed in median, interquartile range, and range. Student’s t-tests were performed to compare blood pressure, serum lipids, blood glucose and hemoglobin levels, while Mann–Whitney U test was performed to compare blood Cd between sexes. Blood Cd was natural log transformed before included in the regression analyses. Multiple regression analyses were performed to identify predictors of CIMT. Statistical significance was considered at a *p* value < 0.05. IBM SPSS statistical software version 25.0 (IBM, US) was used for the analyses.

### Results

#### Characteristics of subjects

Detailed characteristics of the subjects are presented in Table 1. Our subjects were mostly normoweight (42.3%), never smoked (72.4%), non-drinker (98.7%), exposed to secondhand smoke (97.4%) young adults living in Padang, a coastal city as the capital of West Sumatera Province, Indonesia. Parental education, monthly income, and location of residence related to industry were also presented.

**Table 1:** Characteristics of participants (n = 156)

	All		Cd tertile					
			1 (n=52)		2 (n=52)		3 (n=52)	
Sex (n, (%))								
Male	85 (54.5)		28 (53.8)		23 (44.2)		34 (65.4)	
Female	71 (45.5)		24 (46.2)		29 (55.8)		8 (34.6)	
Age (years)								
Mean (SD)	20.7 (1.4)		20.5 (1.2)		20.8 (1.3)		20.9 (1.6)	
Range	18–24							
BMI (n, (%))								
Underweight								
(< 18.5 kg/m^2^)	22 (14.1)		6 (11.5)		7 (13.5)		9 (17.3)	
Normoweight								
(18.5–22.9 kg/m^2^)	66 (42.3)		21 (40.4)		20 (38.5)		25 (48.1)	
Overweight								
(23.0–24.9 kg/m^2^)	28 (17.9)		12 (23.1)		10 (19.2)		6 (11.5)	
Obese								
(> 24.9 kg/m^2^)	40 (25.6)		13 (25.0)		15 (28.8)		12 (23.1)	
Smoking habit (n, (%))								
Never	113 (72.4)		44 (84.6)		45 (86.5)		24 (46.2)	
Former	12 (7.7)		4 (7.7)		4 (7.7)		4 (7.7)	
Current	31 (19.9)		4 (7.7)		3 (5.8)		24 (46.2)	
Secondhand smoking exposure (n, (%))								
Never	4 (2.6)		1 (1.9)		2 (3.8)		1 (1.9)	
Sometimes	81 (51.9)		36 (69.2)		28 (53.8)		17 (32.7)	
Frequently	51 (32.7)		12 (23.1)		20 (38.5)		19 (36.5)	
Always	20 (12.8)		3 (5.8)		2 (3.8)		15 (28.8)	
Alcohol intake (n, (%))								
Yes	2 (1.3)		–		–		2 (3.8)	
No	154 (98.7)		52 (100)		52 (100)		50 (96.2)	
Parental education (n, (%))								
Without college education	56 (35.9)		14 (26.9)		21 (40.4)		21 (40.4)	
College education	68 (43.6)		29 (55.8)		19 (36.5)		20 (38.5)	
Postgraduate education	32 (20.5)		9 (17.3)		12 (23.1)		11 (21.2)	
Parental monthly income (n, (%))								
< 3 million IDR	49 (31.4)		14 (26.9)		17 (32.7)		18 (34.6)	
3–10 million IDR	91 (58.3)		30 (57.7)		33 (63.5)		28 (53.8)	
> 10 million IDR	16 (10.3)		8 (15.4)		2 (3.8)		6 (11.5)	
Residence close to metal workshop or industry (n, (%))								
Yes	23 (14.7)		11 (21.2)		7 (13.5)		5 (9.6)	
No	133 (85.3)		41 (78.8)		45 (86.5)		47 (90.4)	

*SD *= *standard deviation, BMI *= *body mass index, IDR *= *Indonesian Rupiah*

#### Blood pressure, Hb, blood glucose, serum lipids, blood Cd and CIMT

Descriptive statistics of systolic and diastolic blood pressure, Hb, blood glucose, serum lipids, blood Cd, and CIMT are presented in Table 2. Male subjects showed statistically significantly higher systolic blood pressure and Hb levels compared to female subjects (p < 0.001). There were no differences in diastolic blood pressure, blood glucose, total cholesterol, HDL-cholesterol, and LDL-cholesterol levels between sexes. Blood Cd level in young adults of Padang is shown in Table 2 with further comparison between sexes. The median of blood Cd in all subjects was 0.61 μg/L with no statistically significant difference between sexes (p = 0.60). The range of blood Cd value was 0.01–5.96 μg/L. No sample was below the LOQ. CIMT of CCA and ICA were found to be statistically significantly thicker in male compared to female subjects (p < 0.001; Table 2).

**Table 2:** Blood pressure and concentration of haemoglobin, blood glucose, serum lipids, blood Cd and CIMT of young adults from Padang

Variable	All (n = 156)				Male (n = 85)		Female (n = 71)		p-value^#^
	Mean (SD)	Median (IQR)	Min	Max	Mean (SD)	Median (IQR)	Mean (SD)	Median (IQR)	
Systolic blood pressure (mmHg)	115.1 (14.7)		80	167	122.8 (12.9)		105.8 (10.6)		< 0.001
Diastolic blood pressure (mmHg)	71.1 (9.4)		45	107.5	72.4 (10.2)		69.4 (8.2)		0.04
Hemoglobin (g/dL)	14.4 (2.3)		9.1	18.2	15.7 (1.8)		12.8 (1.7)		< 0.001
Blood glucose (mg/dL)	78.5 (8.6)		55.4	97.4	80.3 (8.4)		76.5 (8.4)		0.01
Total cholesterol (mg/dL)	165.8 (29.5)		82.2	267.1	166.2 (29.1)		165.4 (30)		0.88
HDL-cholesterol (mg/dL)	70.9 (22.7)		31.1	173.7	71.6 (21.6)		70.1 (24.1)		0.69
LDL-cholesterol (mg/dL)	71.3 (24.7)		22.7	163.8	70.6 (22.9)		72.1 (26.9)		0.71
Blood Cd (μg/L)	0.94 (1.07)	0.61 (0.7)	0.01	5.96		0.61 (− 0.99)		0.59 (− 0.51)	0.6
Carotid intima-media thickness (CIMT)									
Common carotid artery (CCA) (mm)	0.049 (0.007)		0.033	0.07	0.052 (0.006)		0.045 (0.007)		< 0.001
Internal carotid artery (ICA) (mm)	0.045 (0.007)		0.025	0.06	0.047 (0.006)		0.042 (0.006)		< 0.001

^#^Student’s t test, except for Blood Cd where Mann–Whitney U test was used

*SD* standard deviation, *IQR* interquartile range

#### Predictors of CIMT

To identify factors associated with CIMT, we performed a multiple regression with blood Cd adjusted for age and sex only (Additional file 1: Table  S1), and then we carried out a multivariable model including all relevant variables and blood Cd (Table 3). Our model predicted 21.9% variation in CCA IMT, with sex as the best predictor (β = − 0.438 [95% confidence interval (CI) − 0.662, − 0.214]; p < 0.001). The model for predicting ICA IMT, where 16.5% variation is accounted for, showed that sex was the best predictor (β = − 0.529 [95% CI − 0.761, − 0.297]; p < 0.001). Blood Cd was not a predictor of CCA IMT (adjusted R^2^ = 0.219; β = − 0.101 [95% CI − 0.257, 0.055]; p = 0.203) and ICA IMT (adjusted R^2^ = 0.165; β = − 0.055 [95% CI − 0.217, 0.107]; p = 0.503).

**Table 3:** Predictors of CIMT; results of multiple regression analysis by enter method

	Adjusted R^2^	Predictors	Unstandardized coefficients		Standardized β coefficient	95% CI for β		*p*-value
			B	Standard error		Lower bound	Upper bound	
CCA IMT (µm)	0.219	Constant	39.367	11.538				0.001
		Age (years)	0.612	0.433	0.118	− 0.047	0.283	0.160
		Sex (0 = male, 1 = female)	− 6.210	1.610	− 0.438	− 0.662	− 0.214	<0.001
		BMI (kg/m^2^)	0.160	0.128	0.099	− 0.058	0.256	0.214
		Parental education^a^	0.117	0.733	0.012	− 0.137	0.161	0.873
		Exposure to cigarette smoke^b^	0.909	0.785	0.096	− 0.068	0.260	0.249
		Systolic BP (mmHg)	0.047	0.045	0.097	− 0.087	0.281	0.297
		Total cholesterol (mg/dL)	− 0.026	0.020	− 0.108	− 0.270	0.054	0.189
		HDL− cholesterol (mg/dL)	0.018	0.026	0.058	− 0.106	0.222	0.488
		Haemoglobin (g/dL)	− 0.547	0.299	− 0.177	− 0.368	0.014	0.069
		Blood glucose (mg/dL)	− 0.020	0.066	− 0.025	− 0.188	0.138	0.759
		*ln* Blood Cd	− 0.561	0.438	− 0.101	− 0.257	0.055	0.203
ICA IMT (µm)	0.165	Constant	43.531	11.328				<0.001
		Age (years)	0.476	0.425	0.097	− 0.074	0.268	0.265
		Sex (0 = male, 1 = female)	− 7.127	1.581	− 0.529	− 0.761	− 0.297	<0.001
		BMI (kg/m^2^)	0.165	0.126	0.107	− 0.054	0.268	0.193
		Parental education^a^	− 0.287	0.720	− 0.031	− 0.185	0.123	0.691
		Exposure to cigarette smoke^b^	− 0.912	0.771	− 0.101	− 0.270	0.068	0.239
		Systolic BP (mmHg)	− 0.039	0.044	− 0.085	− 0.275	0.105	0.377
		Total cholesterol (mg/dL)	0.008	0.019	0.035	− 0.131	0.201	0.681
		HDL-cholesterol (mg/dL)	− 0.005	0.026	− 0.016	− 0.176	0.147	0.852
		Haemoglobin (g/dL)	− 0.463	0.294	− 0.158	− 0.356	0.040	0.117
		Blood glucose (mg/dL)	0.045	0.065	0.058	− 0.106	0.223	0.483
		*ln* Blood Cd	− 0.289	0.430	− 0.055	− 0.217	0.107	0.503

*BMI* body mass index, *BP* blood pressure, *CI* confidence interval, *CIMT* carotid intima-medial thickness, *CCA* common carotid artery, *ICA* internal carotid artery, *IMT* intima-medial thickness, *ln* natural log transformed

^a^1 = without college education, 2 = college education, 3 = postgraduate education

^b^1 = never, 2 = sometimes, 3 = frequently, 4 = always

### Discussion

Our study of blood Cd levels in Indonesian young adults living in a coastal city of Padang in West Sumatera Province showed that the median blood Cd levels (0.61 μg/L) is quite low compared to a previous study in adolescents in Iran (~ 10 μg/L (mean); [17]). However, this level was higher than that of 9–10-year-old children in Japan (0.34 μg/L (geometric mean); [18]) and 20–34 year-old general population in the US (0.27 μg/L (geometric mean); [19]). The median of blood Cd in our female subjects is lower than that of 18–24-year-old Norwegian women (0.59 *vs*. 1.43 μg/L; [20]).

Our finding in this small study showed that most subjects are exposed to secondhand smoke. A single cigarette is estimated to contain 12 μg of Cd, an average of 10% is inhaled during smoking [21]. With no known elimination pathway, Cd will accumulate in various tissues, mostly in liver and kidneys [22], and also in aorta [23]. With continuous exposure to cigarettes smoking, our subjects may accumulate higher Cd; hence elevates the risk of adverse health effects. Educational measures on smoking prevention and/or avoiding environmental tobacco smoke should be instigated in young adults and the general population.

Quantitative measurement of intima-medial thickness of large superficial arteries, like carotid and femoral arteries, by using B-mode ultrasonography (USG) is a non-invasive, fast, safe, and highly reproducible method to assess individual CVD risk [24]. Epidemiological studies showed that CIMT is a marker of subclinical atherosclerosis and is associated with conventional risk factors of CVD. CIMT calculation is the most widely used non-invasive atherosclerosis assessment by clinicians and clinical investigators [25].

Carotid artery is elastic and, in healthy young subjects, CIMT consists almost all of medial layer. Normal carotid arterial wall is not influenced by age or sex until around 18 years old; after 18 years, diffuse and progressive thickening takes place in intimal layer [26]. Our subjects’ age ranges from 18 to 24-year-old and it is possible that early process of atherosclerosis has taken place in their arteries. We find that CIMT of male subjects is higher than female subjects and this is in line with the available evidence [27]. A previous study in a cohort of young adults aged 27 to 30 years in Netherland found that marked increased in CCA IMT is associated with unfavorable profile of cardiovascular risk [15]. In this study, we found that sex, rather than blood Cd, as the predictor of CIMT. This might be explained by the relatively low levels of blood Cd in our subjects. Furthermore, considering the relatively young age of our participants, atherosclerosis might have not significantly developed to contribute to CIMT.

## Conclusion

Blood Cd level is not a predictor of CIMT in young adults living in Padang, Indonesia. Regardless, our results are filling the gap of Cd biomonitoring data from developing countries.

## Limitations

- Our study is small in size.
- Our study does not reflect the general population.

## Supplementary information

**Additional file 1: Table S1.** Associations of CIMT and blood Cd adjusted with age and sex; results of multiple regression analysis by enter method.

## Data Availability

The dataset generated and/or analyzed during the current study are available from the corresponding author on reasonable request.

## Abbreviations

BMI: Body mass index
BP: Blood pressure
CCA: Common carotid artery
CDC: The US Center for Disease Control and Prevention
CI: Confidence interval
CIMT: Carotid intima-media thickness
CVD: Cardiovascular disease
Hb: Hemoglobin
HDL: High density lipoprotein
ICA: Internal carotid artery
ICP-MS: Inductively coupled plasma-mass spectrometry
IDR: Indonesian rupiah
IMT: Intima-media thickness
IQR: Interquartile range
LDL: Low density lipoprotein
LOD: Limit of detection
LOQ: Limit of quantification
SD: Standard deviation
