# Supplementary material for: Blood Cd levels and carotid intima-media thickness in young adults living in Padang, Indonesia
Source: BMC Res Notes. 2020 Apr 6;13:202. doi: 10.1186/s13104-020-05042-0 (PMC7137246; doi:10.1186/s13104-020-05042-0)
Supplement: Supplementary file 1 — Additional file 1: Table S1. Associations of CIMT and blood Cd adjusted with age and sex; results of multiple regression analysis by enter method. [file 13104_2020_5042_MOESM1_ESM.docx]

| **Additional Table S1. Associations of CIMT and blood Cd adjusted with age and sex; results of multiple regression analysis by enter method** | | | | | | | | |
| --- | --- | --- | --- | --- | --- | --- | --- | --- |
|  | **Adjusted R^2^** | **Predictors** | **Unstandardized coefficients** | | **Standardized β coefficients** | **95% CI for β** | | ***p*-value** |
|  |  |  | **B** | **Standard error** |  | **Lower bound** | **Upper bound** |  |
| **CCA IMT (µm)** | **0.203** | Constant | 36.131 | 8.496 |  |  |  | <0.001 |
|  |  | Age (years) | 0.708 | 0.399 | 0.136 | -0.015 | 0.287 | 0.078 |
|  |  | Sex (0=male, 1=female) | -5.523 | 1.084 | -0.389 | -0.540 | -0.238 | <0.001 |
|  |  | *ln* Blood Cd | -0.654 | 0.399 | -0.118 | -0.260 | 0.024 | 0.103 |
|  |  |  |  |  |  |  |  |  |
| **ICA IMT (µm)** | **0.164** | Constant | 36.419 | 8.262 |  |  |  | <0.001 |
|  |  | Age (years) | 0.486 | 0.388 | 0.099 | -0.057 | 0.255 | 0.212 |
|  |  | Sex (0=male; 1=female) | -4.919 | 1.055 | -0.365 | -0.520 | -0.210 | <0.001 |
|  |  | *ln* Blood Cd | -0.610 | 0.388 | -0.116 | -0.262 | 0.030 | 0.118 |
| *CI=confidence interval; ln=natural log transformed* | | |  |  |  |  |  |  |
